# Assessment of the distribution, bioavailability and ecological risks of heavy metals in the lake water and surface sediments of the Caohai plateau wetland, China

**DOI:** 10.1371/journal.pone.0189295

**Published:** 2017-12-18

**Authors:** Jing Hu, Shaoqi Zhou, Pan Wu, Kunjie Qu

**Affiliations:** 1 College of Resources and Environment Engineering, Guizhou University, Guiyang, PR China; 2 College of Environment and Energy, South China University of Technology,Guangzhou Higher Education Mega Center, Guangzhoou, PR China; 3 Guizhou Academy of Sciences, Guiyang, PR China; 4 State Key Laboratory of Subtropical Building Sciences, South China University of Technology, Guangzhou, PR China; 5 Key Laboratory of Environmental Protection and Eco-remediation of Guangdong Regular Higher Education Institutions, South China University of Technology, Guangzhou Higher Education Mega Center, Guangzhou, PR China; University of Vigo, SPAIN

## Abstract

In this study, selected heavy metals (Hg, As, Cd, Pb, Cr, Cu and Zn) in the lake water and sediments from the Caohai wetland, which is a valuable state reserve for migrant birds in China, were investigated to assess the spatial distribution, sources, bioavailability and ecological risks. The results suggested that most of the higher concentrations were found in the eastern region of the lakeshore. The concentration factor (CF) revealed that Hg, Cd and Zn were present from moderate risk levels to considerable risk levels in this study; thus, based on the high pollution load index (PLI) values, the Caohai wetland can be considered polluted. According to the associated effects-range classification, Cd may present substantial environmental hazards. An investigation of the chemical speciation suggested that Cd and Zn were unstable across most of the sites, which implied a higher risk of quick desorption and release. Principal component analysis (PCA) indicated that the heavy metal contamination originated from both natural and anthropogenic sources.

## Introduction

Although wetlands are highly productive ecosystems that supply a great amount of goods and services to the populations living in their vicinity, they are also highly sensitive [1]. Wetland environments are usually under pressure from industrial activities, and potentially polluting activities are frequently developed around them, influencing ecosystem services [2].

**Fig 1:**
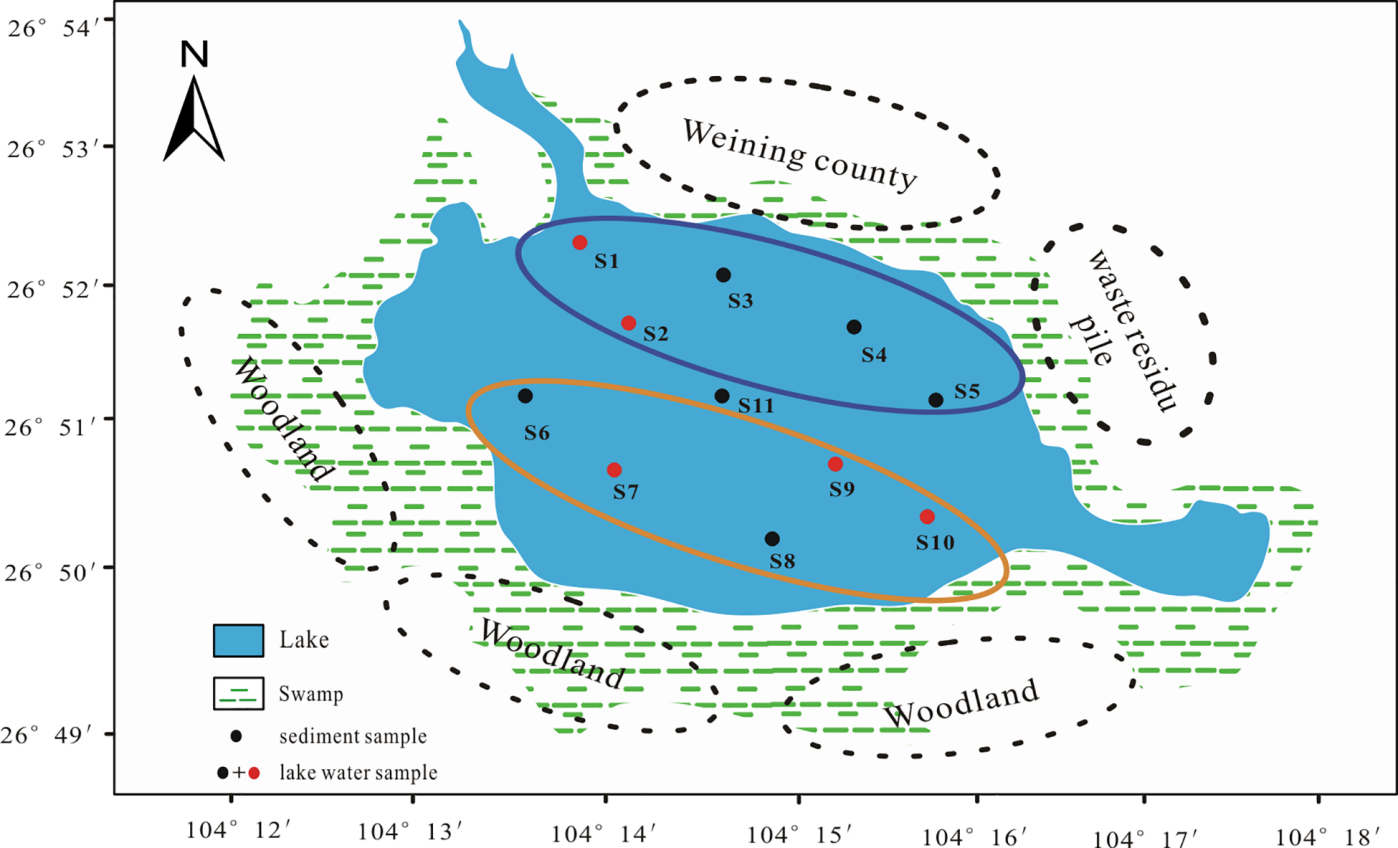
Map of the Caohai wetland and sampling locations.

In this paper, the distribution of heavy metals in the lake water and surface sediments of the Caohai wetland was investigated. The Caohai wetland is situated in northwestern Guizhou, where it is a valuable state reserve for migrant birds in China [3]. The Caohai National Nature Reserve features a temperate climate with an average annual rainfall of approximately 900 mm, an annual average temperature of 10.5°C and an average relative humidity of 79%. Its climate features moderate temperatures in summer and winter, and the humidity is high in summer and autumn and low in winter and spring. There are obvious seasonal differences between the wet and dry seasons [4,5], from May to October, the Caohai wetland enters the wet season. It has an average length of 14.2 km and an average breadth of 1.76 km, and it constitutes a plateau wetland with a water level of approximately 2170 m [6]. The specific bedrock of the region creates alkaline water with an average pH of 8.8 (Fig 1). The water of the Caohai wetland is mainly sourced from precipitation, and the second source is groundwater. The groundwater (Mao Jia Hai Zi river, Dongshan river, Baima river and Dazhong river) that inflows into the Caohai wetland originates from the spring water, the flow of this groundwater depends on the seasonal changes of precipitation to change. In addition, the water of the Caohai wetland flows to the narrow mouth of the northeast to become the headwaters of the Gezhe river [7].

In recent years, the Caohai wetland has been subjected to multiple pollution sources such as zinc smelting activities, especially from Hezhang County, which is located approximately 15 km away [8]. Heavy metals from the smelting waste are released by natural leaching and flow into the Caohai wetland via surface runoff. Weining County is located in the northeast of the Caohai wetland and has many automotive repair facilities, electroplating factories and small livestock farms. Large amounts of industrial wastewater, agricultural effluent and sanitary sewage are discharged into the wetland, causing heavy pressures on the ecological environment of Caohai [9] (Text A in S1 Text, S1 and S2 Figs).

The lake water is the primary receiver of pollution, and heavy metals are widely distributed in it [10]. Meanwhile, it is widely considered that lake sediments are the secondary pollution source restricting the water quality [11,12]. Because heavy metals cannot be purified by the self-purification capacity of water, they are constantly deposited and accumulated into sediments, causing the lake sediments to become a considerable sink for anthropogenic heavy metal emissions [13,14]. However, heavy metals in the sediments were not stable, and with the change in the physical and chemical properties of water, heavy metals could again release and contaminate the overlying water, thus posing a great threat to human health via biological accumulation and the food chain [15,16]. In addition, the content of heavy metals in the sediments could reflect the sustainability of sediments in the overlying water and reflect the degree of danger of the region. Therefore, it is necessary to examine the distribution, sources and ecological risk of heavy metals in the lake water and surface sediments.

In this study, we explored the environmental condition of the Caohai wetland in the context of the ecological risks associated with zinc pollution from smelting activities. Our primary aims were to (1) reveal the distribution of heavy metals in the lake water and surface sediments; (2) evaluate the degree of heavy metal pollution based on sediment quality guidelines, ecological risk indices, concentration factor (CF; [17]) and pollution load index (PLI; [18]); (3) investigate the bioavailability of heavy metals in the sediment by studying chemical speciation; and (4) explore the sources of heavy metals in the Caohai wetland by using principal component analysis (PCA).

## Materials and methods

### Ethics statement

This study did not involve endangered or protected species, no specific permissions were needed for sampling and analysis in study region.

### Sample collection

In our study, lake water and surface sediments (from the top 10 cm) were collected at 11 sites in the wetland in August 2015 (S1 Table). Every site included 3 sampling points (within 5 m*5 m), every point was sampled three times and the average value of the three samples serves as the final display for one site sample. Eleven monitoring sites were divided into three groups. Group 1 (Site 1, Site 2, Site 3, Site 4 and Site 5) was close to the northeast of the Caohai wetland. Group 2 (Site 6, Site 7, Site 8, Site 9 and Site 10) was near the southwest, and Site 11 belonged to Group 3, which was located in the center of the Caohai wetland.

Group 1 included four sites near Weining County (Site 1, Site 2, Site 3 and Site 4) to reflect the input of urban pollution; meanwhile, Site 1 was the outlet of the wetland, and one site (Site 5) was close to the waste residue pile from indigenous zinc smelting. Group 2 (Site 6, Site 7, Site 8, Site 9 and Site 10) was set as a cleaning control group close to the woodland, and Site 7 was a transition area between Group 1 and Group 2.

All containers were pre-cleaned, acid washed (10% HNO_3_) and rinsed with Milli-Q water, followed by oven drying at 40°C. For the water collection, water samples were collected in plastic bottles and then filtered at the scene using a 0.45-μm filter membrane. The pH was adjusted to below 2 using concentrated nitric acid (GR), transferred to a clean microcentrifuge tube with the lid fastened, and transported to the laboratory and preserved in the dark at 4°C in preparation for heavy metal content determination [19].

For the sediment collection, 3 sampling points within 5 m*5 m of each other were repeatedly sampled at each sampling site, and approximately 300 g of sediment was collected at every point. The samples were mixed uniformly, numbered, sealed and placed into zip lock bags in an ice-box, and transferred back to the laboratory [14]. Because underwater sampling should proceed in a stable and careful manner to avoid stirring the sediments, our motions during sampling were slow and controlled. The sediment samples were freeze-dried and mashed with a sterile stick, the gravel was discarded and the sample was ground gently with an agate mortar. It was then sieved through a 200-mesh nylon sieve, packed in a clean plastic bag and sealed for processing.

### Concentration analyses

For the heavy metals analysis, the lake water samples were tested directly. Conversely, sediment samples require a certain amount of preprocessing. For the heavy metal content determination in sediments, 0.1 g of each sample was digested with 2 ml of aqua regia (HNO _3_ /HCl, 1:3 v/v) for 3 h at 110°C [20]. In each digestion step, duplicate samples and reagent blanks were also added. Using Atomic Fluorescence Spectrometry (AFS, Titian, China) analysis, the Hg and As contents were determined. The concentrations of Pb, Cd, Cu, Cr and Zn were determined according to the Atomic Absorption Spectrophotometric method (GB/T7475-1987) using an atomic absorption spectrophotometer (FAAS, Perkin Elmer, USA). The concentrations of total organic carbon (TOC) were analyzed using an element analyzer (Vario EL cube, GRE). The recovery rates were kept at approximately 95%.

### Speciation analysis

To investigate the speciation of heavy metals in sediments, we chose a 3-stage sequential extraction procedure as described in Davidson et al. [21] and Ma et al. [22]. The specific steps were as follows:

Step 1 (Exchangeable fraction): First, 1.00 g of the sample was placed in a 50 mL centrifuge tube and 40 mL of 0.11 M Hac was added, followed by oscillation overnight. Samples were centrifuged, and the supernatant was collected for the analyses.

Step 2 (Reducible fraction): First, 40 mL of 0.5 M NH_2_OH·HCl was added to the residue of step 1, and the pH was regulated to 1.5 using nitric acid and then extracted as above.

Step 3 (Oxidizable fraction): Here, 10 mL of 8.8 M H_2_O_2_ was added to the residue of step 2. To avoid the loss of drastic action, the sample was kept still for 1 h and then evaporated until it was almost dry, after which 50 mL of 1 M NH_4_Ac was added and the sample extracted as above.

Step 4 (Residual fraction): Total heavy metal is determined, excluding the concentrations of step 1, step 2 and step 3.

### Ecological risk analysis

Since the index concentration factor (CF) could express the pollution caused by a single heavy metal [23], we used CF to investigate each pollution condition, which is defined by the following equation:

$$CF=\frac{Me_{sample}}{Me_{baseline}}$$

where Me_sample_ is the measured concentration of metal and Me_baseline_ is the natural abundance of a given heavy metal (Text C in S1 Text). The sediment contamination was classified into 4 grades: low degree (CF < 1), moderate degree (1≤CF < 3), considerable degree (3≤CF < 6) and a very high degree (CF≥6; [19]).

As sediments usually contain combined toxicant groups, such as the combined pollution by heavy metals, pollution by a single heavy metal could therefore not be determined in the sediment. Accordingly, in this study, the pollution load index (PLI) was used to identify the integrated pollution levels [18]. The PLI is defined by the following equation:

$$PLI={\left(CF_{1}\times CF_{2}\times CF_{3}\times \ldots CF_{n}\right)}^{1/n},$$

where CF_n_ is the CF value of metal n.

To assess the potential influence of combined heavy metal pollution, we used the mean effects range-median quotient (mERM-Q), as follows:

$$mERM-Q=(\sum_{i=1}^{n}ERM-Q_{i})/n,$$

$$ERM-Q_{i}=C_{i}/ERM_{i},$$

where C_i_ is the concentration of the selected metal i, ERM_i_ is the ERM of the metal i, and n is the amount of the selected metal. Four grades are classified: mERM-Q≤0.1, representing a low priority site; 0.1<mERM-Q≤0.5, representing a medium-low priority site; 0.5<mERM-Q≤1.5, representing a high-medium priority site; and mERM-Q>1.5, representing a high priority site. These are associated with a 9%, 21%, 49% and 76% probability of being toxic, respectively [24].

## Results and discussion

### Heavy metals in the water of the Caohai wetland

The total contents of mercury (Hg), arsenic (As), cadmium (Cd), lead (Pb), chromium (Cr), copper (Cu) and zinc (Zn) are reported in Table 1. The concentrations revealed significant spatial variation. The metal concentrations in the lake water exhibited wide ranges of 0.029–0.143 μg/l for Hg, 1.346–2.968 μg/l for As, 0.867–3.527 μg/l for Cd, 2.004–5.587 μg/l for Pb, 2.447–5.587 μg/l for Cr, 1.828–2.631 μg/l for Cu and 28.923–55.782 μg/l for Zn. The comparatively high levels of metals in the lake water were generally exhibited in the northeastern sampling sites (e.g., sites 3, 4 and 5), and the metal concentration gradually decreased along the transect from the northeast to southwest, except for Cu, for which the concentrations were similar for the sediments from the northeast (2.304 μg/l on average) and the southwest (2.219 μg/l on average). Sampling sites Site 3 and Site 4, which were located on the eastern coast of the Caohai wetland close to Weining County, indicated the highest concentrations of Hg, Cd, Cr and Zn. Both Site 7 and Site 8 were situated in the southwest of the Caohai wetland, which is farther from the contaminant sources; thus, these sites were less affected by human activities, and exhibited comparatively lower concentrations of metals.

**Table 1:** Heavy metal concentrations in the overlying waters of the Caohai wetland.

Monitoring site		Concentration (μg/l)						
		Hg	As	Cd	Pb	Cr	Cu	Zn
Group 1	Site 1	0.038	1.449	0.867	2.563	4.272	2.067	33.219
	Site 2	0.033	2.329	1.032	6.742	5.587	2.325	40.645
	Site 3	0.142	1.643	3.013	5.583	4.742	2.364	55.782
	Site 4	0.116	2.031	3.527	4.681	4.831	1.937	41.92
	Site 5	0.076	1.938	2.229	2.004	3.567	2.052	39.427
Group 2	Site 6	0.047	2.476	1.162	2.537	2.822	1.828	37.936
	Site 7	0.041	1.346	1.896	2.483	3.092	2.282	28.923
	Site 8	0.029	1.977	0.254	3.013	3.87	2.089	38.714
	Site 9	0.065	2.968	1.731	4.462	2.967	1.987	54.236
	Site 10	0.074	1.762	1.968	2.174	2.447	2.49	39.538
Group 3	Site 11	0.055	1.530	1.654	3.954	4.087	2.631	44.413
SEPA [25]	NSC Class I	0.05	50	1	10	10	10	50
	NSC Class II	0.05	50	5	10	50	1000	1000
	NSC Class III	0.1	50	5	50	50	1000	1000

Compared with the reference values of environmental quality standards for surface water of China [25], As, Pb, Cd and Cu measure up to NSC Class I standard, Cd and Zn only achieve NSC Class II, and Hg belongs to NSC Class III. Thus, the water of the Caohai wetland belongs to NSC Class III, and the national standard prescribes a limit to this type of water for use in aquaculture, swimming or secondary drinking water. With reference to the national standards, there is a comparatively serious Hg pollution and slight Cd and Zn pollution in the Caohai wetland.

**Fig 2:**
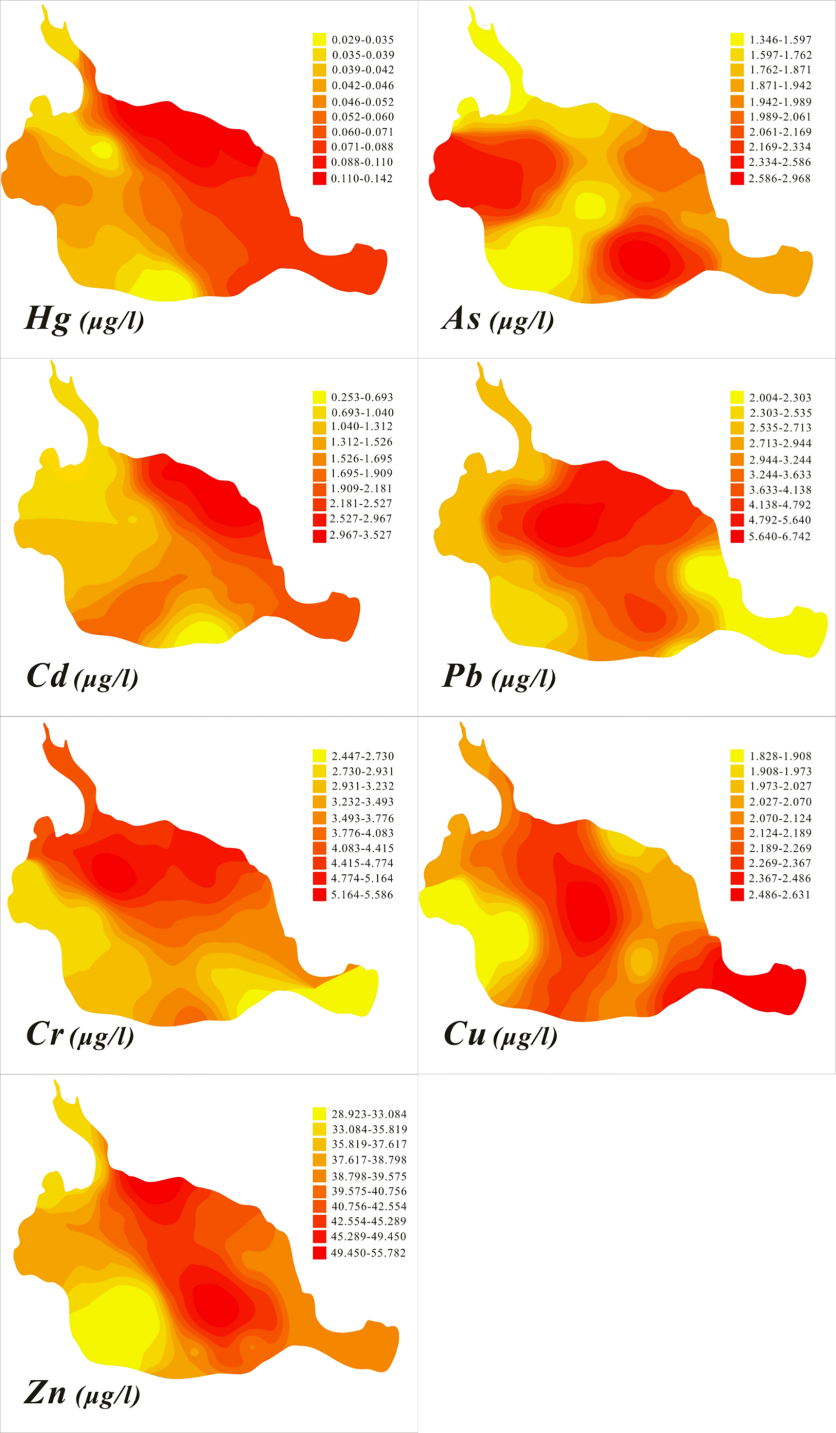
Distributions of heavy metal concentrations in the overlying water.

Although there are significant differences between the northeast and southwest, it is important to quantify the variability and reveal the patterns in the variability. Fig 2 reveals similar distribution patterns of heavy metals in the lake water across the study area and specific analyses are shown in the Text B of SI. The northeastern regions exhibited higher heavy metal concentrations than those in the southwest. Higher concentrations were largely distributed close to Weining County, and lower concentrations were found in areas situated further away from Weining County. This is likely a result of the frequent human activities along the lakeshore and the influence of living wastewater discharge.

### Heavy metals in the sediments of the Caohai wetland

To understand how severe the metal pollution is in this study region, the pollution levels recorded in other areas are provided in Table 2 for comparison. A comparison of data sets revealed that the Cd and Zn levels were much higher than in the other sites listed in Table 2, verifying that it is considerably more prolific in this region. This is most likely because the Hezhang zinc smelting area is situated approximately 15 km from the Caohai wetland and because Cd is usually present in this industry [3]. According to the sediment quality guidelines (SQGs) of China, the heavy metals Hg, Cd and Zn exceed the standard Class I, Zn is above the Class II, and Cd exceeds Class III. It is evident that the study area is contaminated by heavy metals, and furthermore, there appears to be strong enrichment of Zn and Cd. Additionally, using the SQGs from the State Environmental Protection Administration of China (SEPA), Cd was found to be above the threshold level for the heavily polluted category. These results reveal that heavy metal pollution in the Caohai wetland, particularly the sites located in the northeast, have reached the heavily polluted level.

**Table 2:** The Mean concentrations of heavy metals in surface sediments of the Caohai wetland; the related values reported for the surface sediments of other areas; and some standard limit values.

	Concentration (μg/g dry wet)							Reference
	Hg	As	Cd	Pb	Cr	Cu	Zn	
DZB: mean(SD)	0.55(0.40)	16.50(5.15)	12.58(7.90)	32.54(13.00)	38.44(12.46)	21.87(2.09)	386.41(59.89)	Present study
Range (n = 10)	0.17–1.22	8.86–23.57	1.74–23.41	20.04–54.31	24.47–58.63	18.28–23.31	289.23–457.82	
Jiaozhou Bay, China	NA	9.2	0.42	55.2	69.9	38.8	107.4	Xu et al. [26]
Le'an River, China	0.454	32.45	4.713	100.94	62.80	266.40	273.29	Chen et al. [27]
West Bengal, India	NA	5.85	0.14	15.14	40.10	24.20	NA	Antizar et al. [28]
NSC Class I	0.20	20.0	0.5	60	80	35	150.0	SEPA [25]
NSC Class II	0.50	65.0	1.5	130	150	100	350.0	
NSC Class III	1.00	93.0	5	250	270	200	600.0	
ERL	0.15	8.2	1.2	47	81	34	150	Long et al. [29]
ERM	0.71	70	9.6	218	370	270	410	
Non-polluted	≤1.0	<3	-	<40	<25	<25	<90	Perin et al. [30]
Moderately polluted	-	3–8	-	40–60	25–75	25–50	90–200	
Heavily polluted	>1.0	>8	>6	>60	>75	>50	>200	

NA: not available.

### Ecological risks of heavy metals in sediments

**Fig 3:**
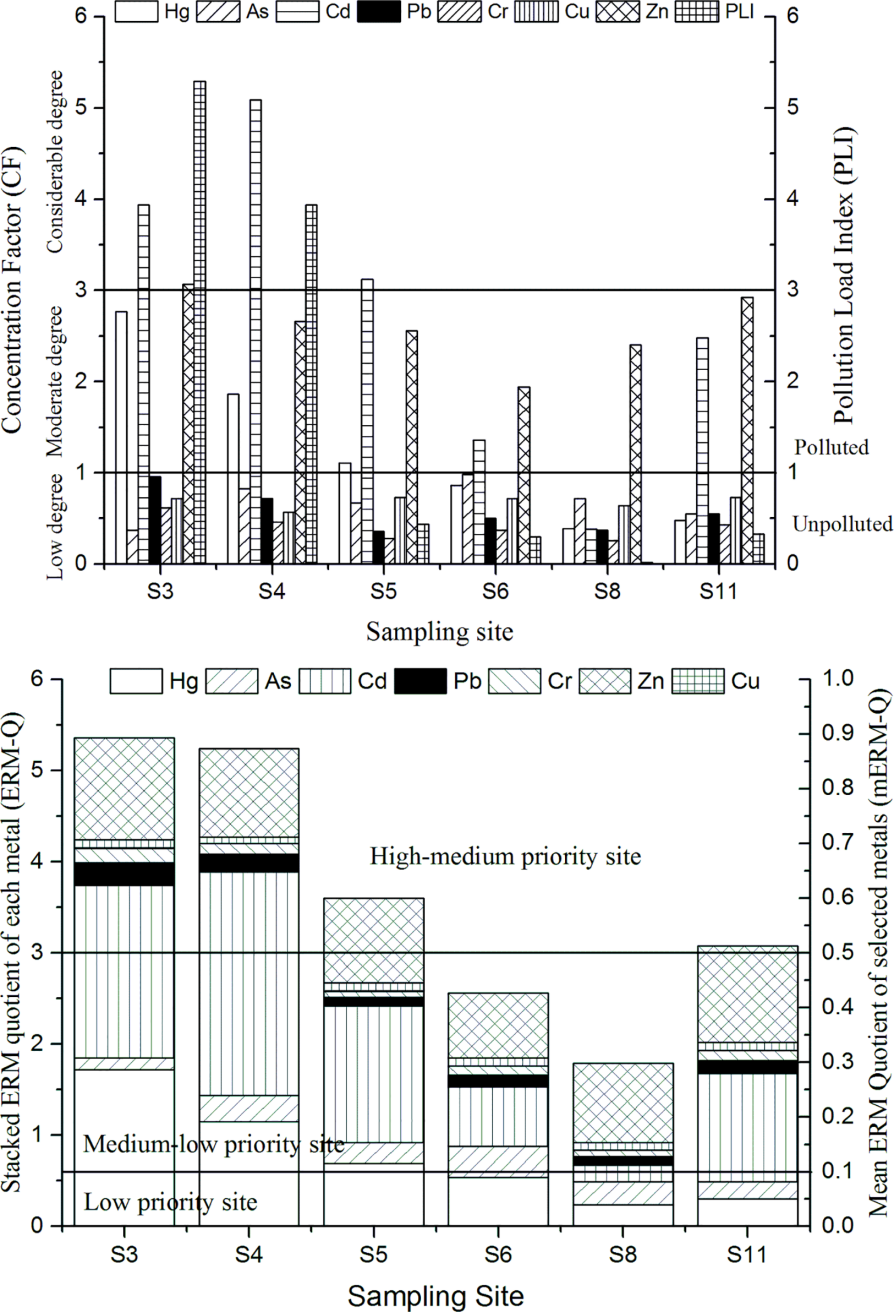
Calculated result of 7 heavy metals in sediments of the Caohai wetland.

Fig 3 lists the CF values and indicates that at most sites, Cd is considerably high, suggesting that the sediments should be marked as polluted by Cd (Site 3 Table). In fact, Cd was the biggest metal pollutant, as its mean and highest CF values were 2.73 and 5.09, respectively. Similarly, Zn and Hg were also high but secondary to Cd, with mean CF values of 2.59 and 1.25, respectively. The CF values of other metals were all <1. Considering the above analyses, it was established that the Caohai wetland sediments were contaminated by Cd, Zn and Hg at varying levels. It is generally accepted that many primary environmental problems in the area are related to mining activities, including the weathering of ore and the discharge from the ore flotation plant [27]. In particular, since it is difficult to control the water seeping from the tailings ponds and non-point sources of pollution, a great variety of pollutants and major heavy metals are potentially discharged into the Caohai wetland.

The PLI values of Site 3 and Site 4 were >1, demonstrating that the Caohai wetland exhibited pollution. Although the PLI values of the other sites (Site 5, Site 6, Site 8 and Site 11) were <1, suggesting that these sites have not been subjected to serious anthropogenic pollution, the heavy metals that originate from anthropogenic sources display higher biological availability than do those from natural sources; hence, the latter might be easier to transfer along the food chain [31]. The PLI values decrease along the northeast near Weining County to the southwestern woodland area. It is evident that anthropogenic heavy metals have increased the PLI values in the study area; Site 3 and Site 4 revealed the highest PLI values, decreasing towards the southwest, where Site 8 recorded the lowest PLI value of 0.02. The PLI values suggest that the order of the combined heavy metal pollution was Site 3 (5.29) > Site 4 (3.94) > Site 5 (0.44) > Site 11 (0.33) > Site 6 (0.30) > Site 8 (0.02). Both the PLI and CF values indicated that the most heavily polluted site was Site 3, for which the heavy metals Cd, Zn and Hg had the greatest contribution (Text D in S1 Text).

SQGs have been developed to assess the chemical sediment contents in the context of conservation and are predictive of adverse effects (USPE). These SQGs have been used to determine the biological effects of sediment pollution in previous research [19]. Following this research, we decided to implement the effects-range-low (ERL) and effects-range-median (ERM) to determine the biological effects of pollution in the Caohai wetland. In this study, the heavy metal Cd exceeded the ERM in our study region, and the most polluted site was determined to be Site 4. Furthermore, Site 3, Site 5 and Site 11 (close to Site 4) all exceeded the ERL. The results indicate that Cd has the greatest impact on biological resources.

The results (Fig 3) suggested that the mERM-Q values of the sediments in the Caohai wetland ranged from 0.25–0.76 and were thus categorized as ‘medium-low priority’ to ‘high-medium priority’ with an 0.1<mERM-Q < 1.5, corresponding to a combined probability of 21 to 49% toxicity (S4 Table). Furthermore, the maximum value for mERM-Q was found at Site 3 in the northeastern Caohai wetland. As it is in the vicinity of Weining County, it might indicate that anthropogenic heavy metals sources display higher toxicity.

### Bioavailability of heavy metals in sediments

**Fig 4:**
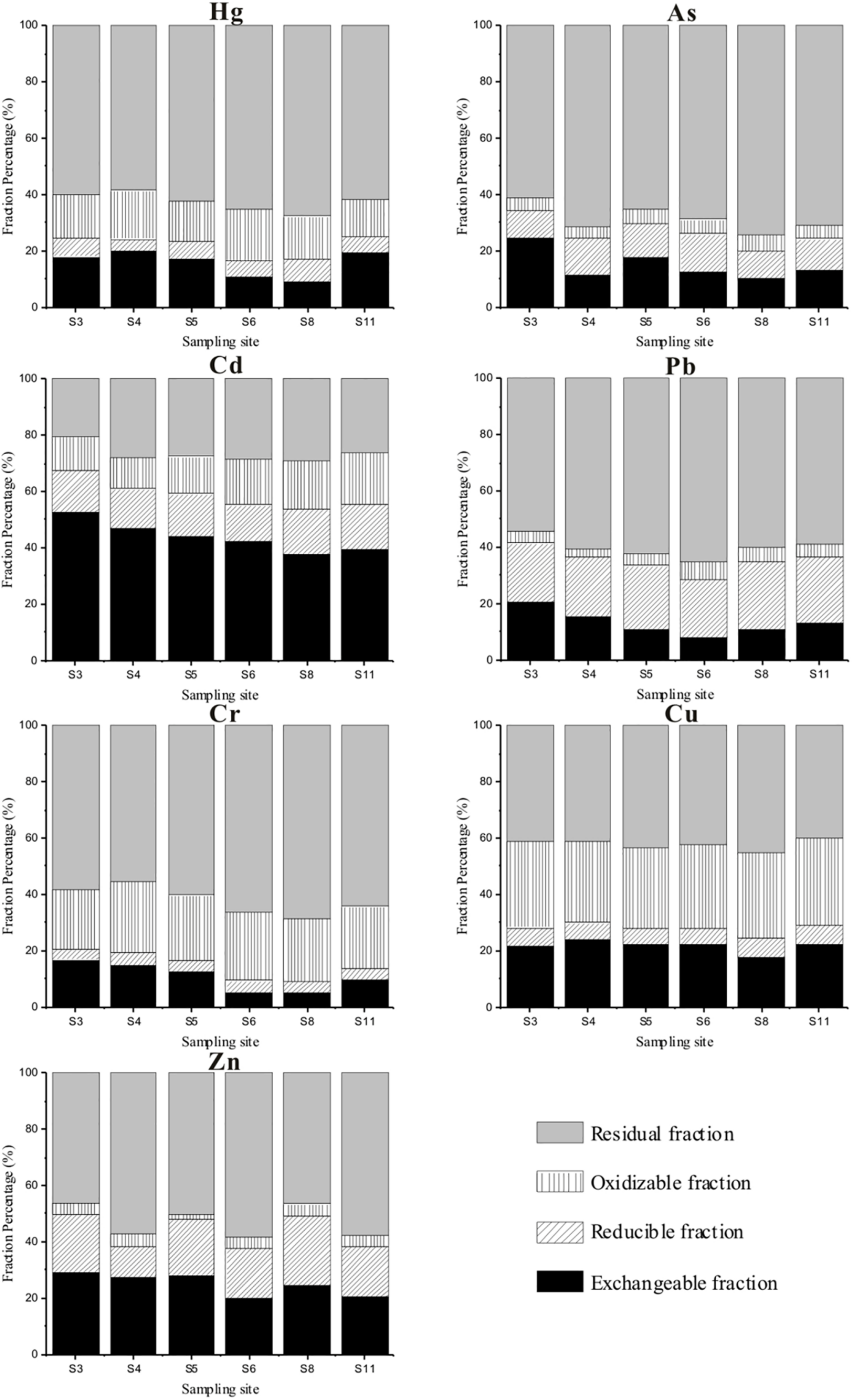
Chemical speciation of heavy metals in sediment.

The chemical speciation of heavy metals could reflect their mobility and bioavailability. We used an optimized BCR (European Community Bureau of Reference) method to determine the chemical speciation of the 7 heavy metals detected in the sediments. Fig 4 indicates the differences in chemical speciation of each heavy metal and reveals significant spatial variation among the heavy metals as a result of multiple factors.

Sites 3 and 4 exhibited nearly the highest exchangeable fractions, and the lowest was detected at Site 8. The position of sites Site 3 and Site 4 in the northeast of the wetland near Weining County renders them susceptible to sources of high-bioavailability heavy metals. Cadmium and Zn exhibited the highest exchangeable fractions, with mean values of 43.69% and 24.86%, respectively. These represent the exchangeable fractions with the strongest toxicity that could be readily biologically absorbed and released under conditions of neutral and weak acids. In this study, the pH of the Caohai wetland was between 7.42 and 9.21, with an average of 8.25, indicating weak alkalinity. Although this environment is not conducive to the release of exchangeable metals under normal conditions, the exchangeable metals in sediments have the risk of secondary release should they be impacted by acid rain or aquatic plant decay. Some research has shown that acid rain frequently occurs in the region of Guizhou province [32–34]. The acid rain frequency was highest in the winter, accounting for 43.0% of the whole year, followed by spring (35.5%), and January had the lowest pH value (5.26~5.67) [33]. Thus, the exchange of metals in the Caohai wetland was perfectly possible via secondary release in the case of acid rain in winter and should be given attention.

The reducible fractions usually accumulate by adsorption or precipitation. When the redox potential decreases or dissolved oxygen is lacking in water, the reducible fractions are released, which may result in water pollution. In this research, Pb had the highest reducible fraction (22.24%), whereas Cr, Cu and Hg had the lowest reducible fractions of 4.10%, 6.04% and 6.25%, respectively. The other metals ranged from 11% to 18%. Domestic sewage, which is potentially input into the wetland from Weining County, includes oxygen-consuming organics, which could lead to a potential oxidation reduction. Thus, the sediments in the northeast of the wetland are at risk of released Pb.

Oxidable fractions are centered on heavy-metal ions, and the combination of active groups of organic ligands or heavy metals generate substances that are insoluble in water. In this research, the contents of organic matter in the Caohai wetland varied from 6.28~47.29%, with an average of 24.38%. Copper and Cr had the highest oxidable fractions (29.98% and 23.08%), whereas Zn, As and Pb had the lowest oxidable fractions of 3.86%, 4.63%% and 4.90%, respectively. Oxidable fractions break down under strong oxidation conditions; thus, the oxidable fractions of the heavy metals in the Caohai wetland could not easily be released under normal moderate to weak oxidation conditions. Copper and Cr had the highest oxidable fractions and had a high affinity with organic matter and sulfide.

Residual fractions are often believed to occur in the original and secondary ore mines, exhibit a high degree of stability and are rarely utilized by animals. The values of the residual fractions exhibited wide ranges of 58.58%~67.75% for Hg, 61.27%~74.32% for As, 20.32%~29.18% for Cd, 54.24%~65.38% for Pb, 55.36%~68.64% for Cr, 39.99%~45.21% for Cu and 46.47%~58.21% for Zn. The lower the residual fraction is, the easier it is to release and cause secondary pollution. Therefore, the order of the bioavailability of heavy metals is Cd>Cu>Zn>Pb>Cr>Hg>As.

### Source identification of heavy metals in sediments

The sources of heavy metal pollution of sediments are both natural and anthropogenic [35]. Thus, due to their biological toxicity, the geochemical properties and transfer ability of heavy metals could be influenced by TOC [36]. Considering Pearson correlation matrix could reflect the degree of linear association of two variables and support the results obtained by PCA/FA [37], we used the Pearson correlation matrix to investigate the relationship between TOC and heavy metals (Table 3) to understand the characteristics of the metals in the Caohai sediments. The results indicated that the TOC content was highly significantly positively correlated with As (at the 0.01 level). This is due to the chemical constitution of the sediments reflecting the coupling interaction between the cycles of heavy metals and TOC [22]. Meanwhile, the highly significantly positive correlation implied that the distribution of As was under the influence of TOC rather than an anthropogenic source, and combined with the analysis of distribution, quantified the variability (in SI) and suggested that the main source of arsenic is natural sources. Instead, TOC content was negatively correlated with other heavy metals, which showed that these heavy metals were influenced by anthropogenic sources; in particular, Cr (at the 0.05 level), Cu (at the 0.05 level) and Zn (at the 0.01 level) had highly significantly negative correlations. Furthermore, in addition to Cu, other heavy metals (Hg, Cd, Pb, Cr, and Zn) all had highly positive correlations, which might indicate that Cu had a source other than the anthropogenic source. Only Cr was not negatively correlated with Cu, which may suggest that Cr and Cu had the same anthropogenic source input (Text E in S1 Text).

**Table 3:** The correlation of TOC and heavy metals in sediments of the Caohai wetland (N = 18).

	TOC	Hg	As	Cd	Pb	Cr	Cu	Zn
TOC	1							
Hg	-0.275	1						
As	0.904**	-0.358	1					
Cd	-0.197	0.761**	-0.237	1				
Pb	-0.269	0.852**	-0.346	0.689**	1			
Cr	-0.588*	0.694**	-0.535*	0.575*	0.815**	1		
Cu	-0.573*	-0.169	-0.336	-0.294	-0.249	0.197	1	
Zn	-0.599**	0.552*	-0.738**	0.593**	0.592**	0.494*	-0.158	1

**Correlation is significant at the 0.01 level (2-tailed).

*Correlation is significant at the 0.05 level (2-tailed).

To determine the sources of the 7 heavy metals present in Caohai sediments, we used the multivariate principal component analysis (PCA). Using SPSS (19.0), the resulting KMO value was 0.648 and Bartlett's result was 123.396 (df = 28, p<0.001).

Together, the 2 principal components represent 80.1% (PC1 54.5%; PC2 25.6%) of the total variance. As indicated in S3 Fig, the PCA divided the TOC and the 7 heavy metals into 3 groups: Group 1 (Hg, Cd and Pb), Group 2 (As, TOC) and Group 3 (Cu, Cr and Zn). Interestingly, as found in the Pearson correlation matrix, As and TOC formed a group on one side of the plot, distant from other heavy metals (S3 Fig), suggesting that there are significantly different sources in Group 2. Six heavy metals, especially Pb, were positively associated with PC1. The weight of 0.843 on PC1 indicated that the major sources of Pb are related to the discharge from the burning of coal, fuels and leaded petrol into meteoric water [22,38]. Thus, PC1 is primarily of an anthropogenic source. The energy structure is relatively backward in Weining county, and coal burning is still the main source of heat for cooking and warming. Therefore, artificial coal burning might be the primary source of Pb. It was also reported that there is a long history of Zn smelting in Hezhang County, which is located approximately 15 km from the Caohai wetland, and that because Cd and Hg are usually present in Zn ores [3,39], this suggests that the source of Cd, Hg and Pb in Group 1 is probably from mining activity and ore burning. Zn not only came from mining activity but also came from industrial activities. It is well documented that Cu, Cr and Zn are present in metal smelting and electroplating [40], which suggests that the source of Cu, Cr and Zn in Group 3 might originate from industrial activities. Local automobile repair plants and electroplating factories were probably the primary sources of Cu, Cr and Zn.

PC2 is deemed to primarily reflect a natural geological source. Based on the distribution and correlation analysis, As seems to come from natural sources. Meanwhile, Pan et al. [19] suggested that the source of Cd was influenced by the contribution of parent rocks; thus, Cd also has a natural geological source, whereas Cd has a relatively high loading weight (0.464) on PC2. Hence, PC2 might indicate a natural geological origin for the metal pollution.

Overall, Group 1 was the result of both natural geological and anthropogenic sources, but primarily anthropogenic. Group 2 was primarily due to natural geological sources, whereas Group 3 mostly came from anthropogenic sources.

## Study limitations

This study only determined the concentrations of water samples and sediment samples in the Caohai wetland in August 2015 and used the data to assess the distribution, sources, bioavailability and ecological risks of heavy metals, some problems still need to be further investigated (Text F in S1 Text). In future studies, seasonal variation, effects of water chemistry, sampling size and biological samples should be paid more attention.

## Conclusions

Systemic geochemistry analyses were implemented in the Caohai wetland to investigate the distribution of heavy metals in the lake water and surface sediments. The results revealed, in different media (water and sediments), that heavy metals were found to be present at varying concentrations. With reference to national standards, Hg is the most serious pollutant in water, followed by Cd and Zn, and made the water of Caohai wetland belong to NSC Class III. In sediments, Cd was the most polluted metal and was beyond the limit value of NSC Class III.

Furthermore, the heavy metals were temporally separated, in that the highest heavy metal concentrations were predominantly associated with the northeast, with a general decreasing trend towards the southwest.

The index concentration factor (CF) values suggest that the heavy metals Hg and Zn have moderately polluted the sediments of the Caohai wetland, but the Cd pollution is considerably higher. In addition, the PLI results indicated that the CF values reveal higher metal concentrations in the northeast. Based on the SQGs of the study areas, Cd exceeded ERM and is likely the cause of adverse biological effects, especially at sites 3 and 4. In conjunction with mERM-Q, these results indicate that the combination of these heavy metals in sediments represent a 21% to 49% chance, respectively, of being toxic.

The chemical speciation analysis indicates that Cd and Zn were unstable in most of the study sites, suggesting a higher risk of quick desorption and release, particularly in the case of acid rain in winter. In some districts, especially the northeast, Pb might be released with domestic sewage discharge, thus posing a chronic risk of toxicity for benthonic organisms.

In addition, PCA suggested that in addition to potential natural contributions, the sediments may be influenced by anthropogenic pollution sources, such as coal burning, mining activity, industrial emissions and living wastewater. Therefore, controlling pollutants, improving wastewater treatment and strengthening the supervision and management in the vicinity of the wetland may be important for treating heavy metal pollution. Furthermore, a comprehensive method of precise environmental risk evaluation should be implemented by using Cd, Hg and Zn as the key targets for environmental management and pollution prevention in the Caohai wetland.

## Supporting information

S1 Text (DOCX)

S1 Table Coordinates of sampling points. (DOCX)

S2 Table The concentrations of heavy metals in sediments. (DOCX)

S3 Table CF of heavy metals in sediments. (DOCX)

S4 Table mERM-Q values of the sediments and the probabilities of being toxic(POBT). (DOCX)

S1 Fig Weining county. (PDF)

S2 Fig The southeast of Caohai wetland. (PDF)

S3 Fig The PCA Loading Plot for TOC and Heavy Metals in Sediment. (PDF)
