# Supplementary material for: Assessment of the distribution, bioavailability and ecological risks of heavy metals in the lake water and surface sediments of the Caohai plateau wetland, China
Source: PLoS One. 2017 Dec 18;12(12):e0189295. doi: 10.1371/journal.pone.0189295 (PMC5734908; doi:10.1371/journal.pone.0189295)
Supplement: S1 Text — (DOCX) [file pone.0189295.s001.docx]

**Text A:**

In this study, every site include 3 sampling points (within 5 m*5 m), every points sampling three times on the same day and average value of three samples serve as the final display one site sample. Thus, there were 33 sampling points and 99 samples for water, 18 sampling points and 54 samples for sediment in fact.

The sampling sites had been set in order to investigate the heavy metal pollution of Caohai wetland under different land use patterns. Such as S3 and S4 near the county, S5 close to farmland and the wastes residue pile of indigenous zinc smelting, S6, S7 and S8 were set to compare with S3, S4, S5 respectively for comparison. Meanwhile, S6, S7 and S8 in the nearby of woodland. S1 sites was set at the outlet of the wetland to investigate the pollution emissions of Caohai wetland. Other sites (S2, S9, S10 and S11), set as transition zone from northeast to southwest, were located on the centre line of the wetland, to research the distribution of heavy metal in Caohai wetland.

Weining county is located at northeast of Caohai wetland, where has a population of 200,000. Energy structure is relatively backward in Weining county, coal burning is still the main means of cooking and warming. Since coal containing heavy metals, coal burning might be the one of the sources of heavy metal. On the other hand, there are a few automobile repair plant and electroplating factory scattered across the northeast coast of wetland. Meanwhile, over here, industrial park and port facilities are is under construction, there are bound to cause pollution. Furthermore, discharge of domestic sewage also put heavy burden on Caohai environment.

Indigenous zinc smelting in Weining county has a long history. There are some wastes residue pile of indigenous zinc smelting on the bank near S5. Heavy metals of smelting waste might release by the process of natural leaching and flowed into Caohai wetland by surface runoff.

In the southeast bank of Caohai wetland, there is a tract of woodland with a good ecological environment.


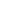


**Text B:**

The content of Hg in the northeast varied between 0.038 and 0.142 μg/l, while in the southwest it varied between 0.029 and 0.074 μg/l. The maximum level was observed at S3 (1.42 μg/l) and the minimum level at S8 (0.29 μg/l). Arsenic exhibited contrary behavior, in that higher contents were found in the southwest at sites S9 (2.968 μg/l) and S6 (2.467 μg/l). This may be due to the fact that As is a naturally occurring metal and is widely distributed in the environment (Zhuang et al., 2015). The As values ranged from 1.449 to 2.329 μg/l in the northeast and 1.346–2.968 μg/l in the southwest. The highest Cd content was also exhibited at site S4 in the northeast and the average value was 40.7% higher than in the southwest. Chromium and its compounds have a wide range of applications in metallurgy, electroplating, leather, paint and other industries (Lin et al., 2013), thus industrial pollution in the northeast coast was the possible cause of Cd distribution.

The highest concentrations of Pb were also reported in the northeast of the Caohai wetland, including at S2 (6.742 μg/l), S3 (5.583 μg/l) and S4 (4.681 μg/l), ranging from 2.004 to 6.742 μg/l. The highest Cr contents were generally found in northeast (S2, S3 and S4). Copper, as a naturally existing metal usually observed in the lithosphere and rivers, may be derived from both natural and anthropogenic sources (Fisher et al., 2016). The highest content of Cu (2.631 μg/l) was found at S11 in the center of the Caohai wetland and its [concentration](http://cn.bing.com/dict/clientsearch?mkt=zh-CN&setLang=zh&form=BDVEHC&ClientVer=BDDTV3.5.0.4311&q=%E6%B5%93%E5%BA%A6)s ranged from 1.828 to 2.631 μg/l. The highest amount of metal detected in this study was Zn, probably as it exists in [great](http://cn.bing.com/dict/clientsearch?mkt=zh-CN&setLang=zh&form=BDVEHC&ClientVer=BDDTV3.5.0.4311&q=%E5%A4%A7%E9%87%8F%E5%AD%98%E5%9C%A8) [quantities](http://cn.bing.com/dict/clientsearch?mkt=zh-CN&setLang=zh&form=BDVEHC&ClientVer=BDDTV3.5.0.4311&q=%E5%A4%A7%E9%87%8F%E5%AD%98%E5%9C%A8) in nature as well as in industrial and mining waste waters ([Qi et al., 2010](#_ENREF_15)). The Zn contents varied from 33.219 to 55.782μg/l in the northeast and 28.923–54.236 μg/l in the southwest, and the highest concentrations were recorded at S3.

**Text C:**

The value of Me_baseline_ came from *Elements background value of China soil ,* Consulted the A layer of soil, the value (mg/kg) were 0.44 for Hg, 24 for As, 4.613 for Cd, 56.32 for Pb, 95.3 for Cr, 32 for Cu and 149.25 for Zn.

**Text D:**

As sediments usually contain combined toxicant groups, such as the combined pollution by heavy metals. Accordingly, in this study, the pollution load index (PLI) was used to identify the integrated pollution levels.

Pollution load index was an evaluation method which established by Tomlinson in order to assess pollution levels of heavy metals. This method could intuitive reflect the contribute to pollution of various heavy metals and the variation trend of heavy metals in space. Moreover, PLI due to convenient and reliable accuracy has been used in many researches (Pan et al., 2014; Lai et al., 2013; Liu et al., 2011). So PLI had been chosen in this study to identify the integrated pollution levels.

In this study, PLI was used to study the degree of pollution in different areas of wetland and reveal the contribution of each heavy metal.

**Text E:**

From the perspective of geological structures, Caohai wetland is epsilon-type structure located in the west wing reflex arc of western Guizhou, north bend the top part of Weining county’s back. From the view of geomorphology, Caohai wetland is the dramatic undulating mountain valley in the plateau and appears ladder-like plateau mountain landforms. The west, south and east of wetland are relatively high, progressively decrease from the centre towards the north. This makes it easy to outflow the pollutant, rather than accumulation of pollutants. If there is no continuous input of pollutants, the self-purification capacity of water environment of Caohai wetland could change the status quo toward a better direction.

However, in recent years, industrial park and port facilities are is under construction would be bound to bring more pollution to wetland. Combining the analysis of the article, the heavy metals, which source from industrial will cause more pollution, such as Cu, Cr and Zn. Instead, the pollution of the heavy metal of natural source could reduced gradually by the self-purification capacity of water environment.

**Text E:**

The clarification and explanation to study limitations.

1. Why not considering the influence of sampling depth

Answer: Caohai wetland is not deep and its average depth is 1.35m. So there is almost no difference in the depth among the sampling sites

1. The total number of samples is insignificant (n = 11 for water and sediment) to understand the overall distribution characteristics of heavy metal in the wetland.

Answer: On one hand, in this study, every point include 3 points in 5 m*5 m, that is to say we make 3 points combine into 1 point, so there are 33 points in fact. Meanwhile, sampling three times in each sampling point and then mixed uniformly as one sample, average value of three samples serve as the final display one sample. On the onther hand, the surface sediments of other studies also did not have too much sampling sites, about a ten, in a similar situation to this study. Second, most studies are like this ( Pan’s study sampling sites= 10; Ma’s study sampling sites= 13; Song’s study sampling sites=9 ).

1. The effect of timing and duration of the rainy season

Answer: Caohai wetland indeed has obvious seasonal differences between the wet and dry seasons. From May to October, Caohai wetland enters wet seasons. However, the average annual rainfall of Caohai wetland is not quite large, only about 900 mm, and no windy and heavy rain during sampling, the lake water is placid.

1. For the top paragraph, some discussion of seasonal impacts is needed.

Answer: The response of manuscript is focused on a period of time to assessment of the distribution, sources, bioavailability and ecological risks of heavy metals. We will store temporal data to enrich the research in future.

1. Bio-monitoring approach to evaluate bioaccumulation and associated impacts on aquatic organisms

Answer: That is actually a very good guidance. However, we are very sorry that we neither collected biologic samples nor used a bio-monitoring approach in this study. We tried to use the concentration, distribution, chemical form and some analysis methods to reveal pollution status, biological toxicity and potential sources. Bio-monitoring approach is the next research direction.
