# Supplementary material for: Assessment of the distribution, bioavailability and ecological risks of heavy metals in the lake water and surface sediments of the Caohai plateau wetland, China
Source: PLoS One. 2017 Dec 18;12(12):e0189295. doi: 10.1371/journal.pone.0189295 (PMC5734908; doi:10.1371/journal.pone.0189295)
Supplement: S1 Table — (DOCX) [file pone.0189295.s002.docx]

**S1 Table .** Coordinates of sampling points

|  | longitude | latitude |
| --- | --- | --- |
| S1 | 104.236 | 26.873 |
| S2 | 104.240 | 26.863 |
| S3 | 104.247 | 26.871 |
| S4 | 104.257 | 26.864 |
| S5 | 104.263 | 26.855 |
| S6 | 104.234 | 26.855 |
| S7 | 104.239 | 26.846 |
| S8 | 104.249 | 26.838 |
| S9 | 104.255 | 26.846 |
| S10 | 104.260 | 26.839 |
| S11 | 104.248 | 26.855 |
