# Supplementary material for: Assessment of the distribution, bioavailability and ecological risks of heavy metals in the lake water and surface sediments of the Caohai plateau wetland, China
Source: PLoS One. 2017 Dec 18;12(12):e0189295. doi: 10.1371/journal.pone.0189295 (PMC5734908; doi:10.1371/journal.pone.0189295)
Supplement: S2 Table — (DOCX) [file pone.0189295.s003.docx]

**S2 Table .** The concentrations of heavy metals in sediments

|  | Hg | As | Cd | Pb | Cr | Cu | Zn |
| --- | --- | --- | --- | --- | --- | --- | --- |
| S3 | 1.22 | 8.86 | 18.17 | 54.31 | 58.62 | 23.08 | 457.82 |
| S4 | 0.82 | 20.01 | 23.49 | 40.43 | 43.59 | 18.28 | 396.52 |
| S5 | 0.49 | 16.17 | 14.38 | 20.04 | 26.97 | 23.24 | 381.69 |
| S6 | 0.38 | 23.57 | 6.26 | 28.16 | 35.65 | 22.97 | 289.23 |
| S8 | 0.17 | 17.23 | 1.74 | 21.08 | 24.47 | 20.32 | 357.48 |
| S11 | 0.21 | 13.17 | 11.43 | 31.19 | 41.33 | 23.31 | 435.71 |
