# Supplementary material for: Assessment of the distribution, bioavailability and ecological risks of heavy metals in the lake water and surface sediments of the Caohai plateau wetland, China
Source: PLoS One. 2017 Dec 18;12(12):e0189295. doi: 10.1371/journal.pone.0189295 (PMC5734908; doi:10.1371/journal.pone.0189295)
Supplement: S3 Table — (DOCX) [file pone.0189295.s004.docx]

**S3 Table .** CF of heavy metals in sediments

|  | Hg | As | Cd | Pb | Cr | Cu | Zn | PLI |
| --- | --- | --- | --- | --- | --- | --- | --- | --- |
| S3 | 2.77 | 0.37 | 3.94 | 0.96 | 0.62 | 0.72 | 3.07 | 5.29 |
| S4 | 1.86 | 0.83 | 5.09 | 0.72 | 0.46 | 0.57 | 2.66 | 3.94 |
| S5 | 1.11 | 0.67 | 3.12 | 0.36 | 0.28 | 0.73 | 2.56 | 0.44 |
| S6 | 0.86 | 0.98 | 1.36 | 0.50 | 0.37 | 0.72 | 1.94 | 0.30 |
| S8 | 0.39 | 0.72 | 0.38 | 0.37 | 0.26 | 0.64 | 2.40 | 0.02 |
| S11 | 0.48 | 0.55 | 2.48 | 0.55 | 0.43 | 0.73 | 2.92 | 0.33 |
