# Supplementary material for: Assessment of the distribution, bioavailability and ecological risks of heavy metals in the lake water and surface sediments of the Caohai plateau wetland, China
Source: PLoS One. 2017 Dec 18;12(12):e0189295. doi: 10.1371/journal.pone.0189295 (PMC5734908; doi:10.1371/journal.pone.0189295)
Supplement: S4 Table — (DOCX) [file pone.0189295.s005.docx]

**S4 Table .** mERM-Q values of the sediments and the probabilities of being toxic(POBT)

|  | ERM-Qi | | | | | | | mERM-Q | POBT |
| --- | --- | --- | --- | --- | --- | --- | --- | --- | --- |
|  | Hg | As | Cd | Pb | Cr | Cu | Zn |  |  |
| S3 | 1.72 | 0.13 | 1.89 | 0.25 | 0.16 | 0.09 | 1.12 | 0.76 | 49% |
| S4 | 1.15 | 0.29 | 2.45 | 0.19 | 0.12 | 0.07 | 0.97 | 0.75 | 49% |
| S5 | 0.69 | 0.23 | 1.50 | 0.09 | 0.07 | 0.09 | 0.93 | 0.51 | 49% |
| S6 | 0.54 | 0.34 | 0.65 | 0.13 | 0.10 | 0.09 | 0.71 | 0.36 | 21% |
| S8 | 0.24 | 0.25 | 0.18 | 0.10 | 0.07 | 0.08 | 0.87 | 0.25 | 21% |
| S11 | 0.30 | 0.19 | 1.19 | 0.14 | 0.11 | 0.09 | 1.06 | 0.44 | 21% |
