# Supplementary figures and images for: Assessment of the distribution, bioavailability and ecological risks of heavy metals in the lake water and surface sediments of the Caohai plateau wetland, China
Source: PLoS One. 2017 Dec 18;12(12):e0189295. doi: 10.1371/journal.pone.0189295 (PMC5734908; doi:10.1371/journal.pone.0189295)

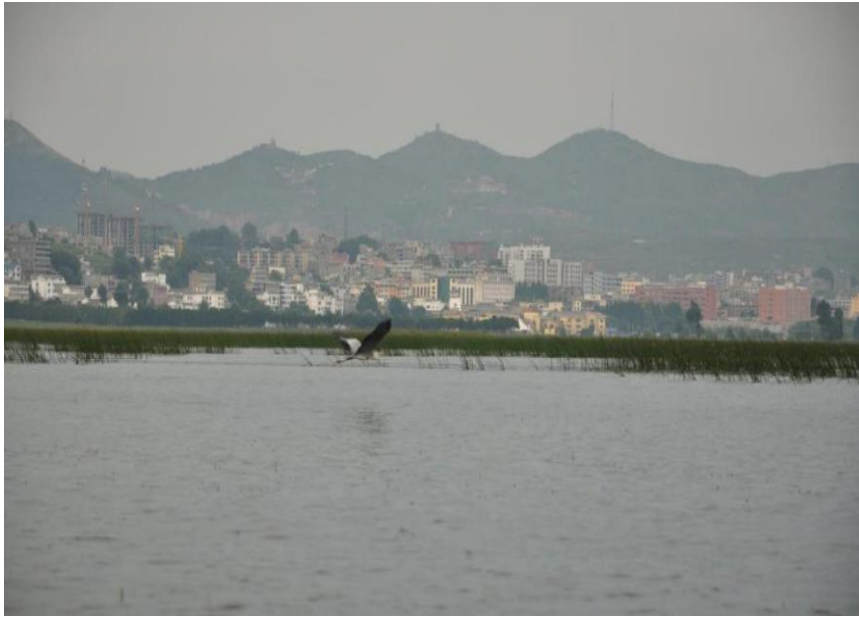

**S1 Fig .** Weining county

Supplement: S1 Fig — (PDF) [file pone.0189295.s006.pdf]

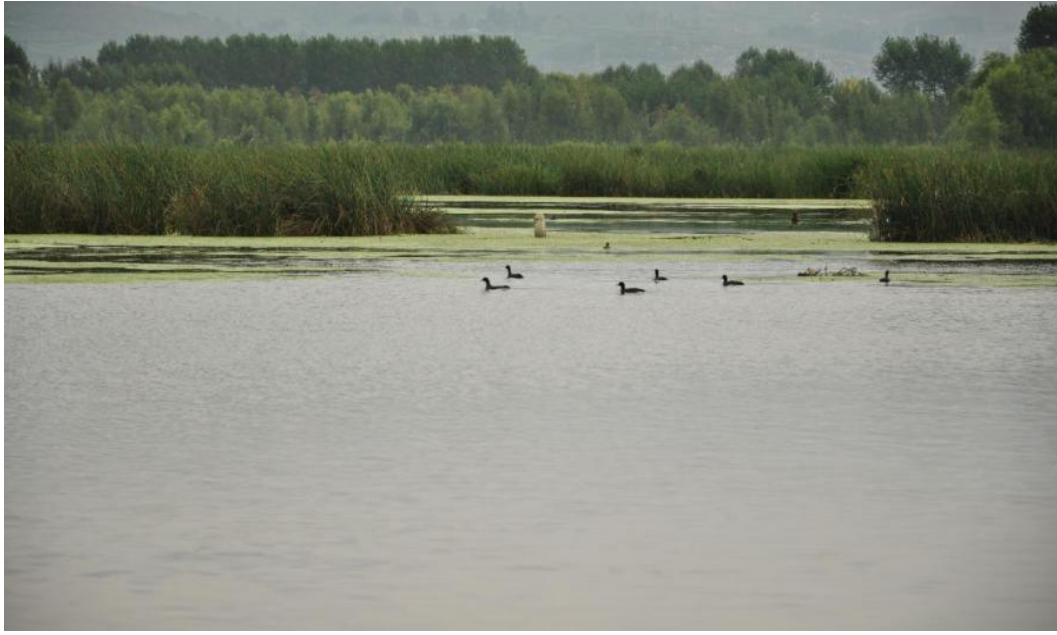

**S2 Fig .** The southeast of Caohai wetland.

Supplement: S2 Fig — (PDF) [file pone.0189295.s007.pdf]

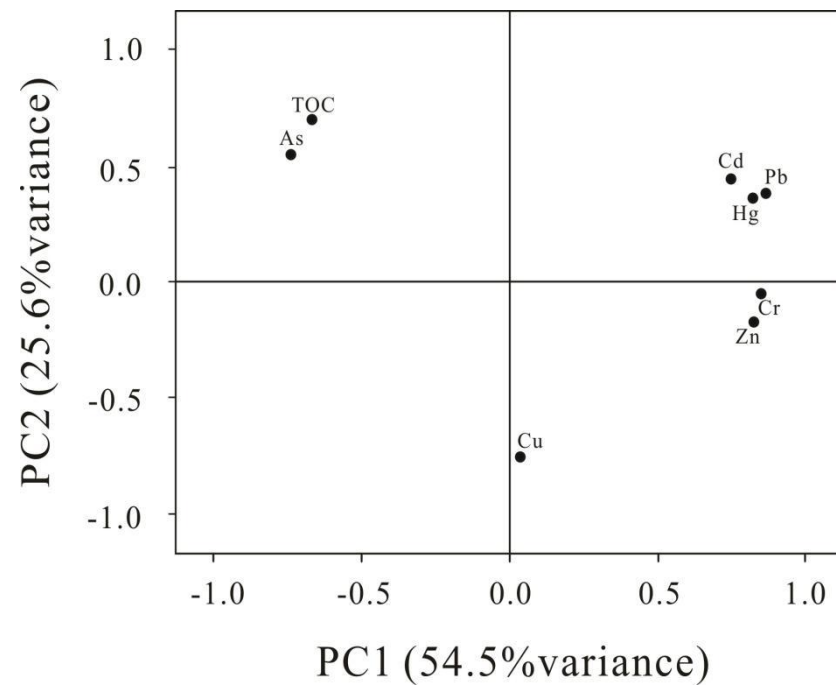

**S3 Fig .** The PCA Loading Plot for TOC and Heavy Metals in Sediment.

Supplement: S3 Fig — (PDF) [file pone.0189295.s008.pdf]
